# Laparoscopic right hemicolectomy for an ascending colon cancer patient with an implantable left ventricular assist device: a case report

**DOI:** 10.1186/s40792-020-01064-9

**Published:** 2020-11-09

**Authors:** Taiki Kajiwara, Takeshi Naitoh, Yusuke Suzuki, Atsushi Kohyama, Hideaki Karasawa, Hideyuki Suzuki, Masatoshi Akiyama, Yoshikatsu Saiki, Kazuhiro Watanabe, Shinobu Ohnuma, Takashi Kamei, Michiaki Unno

**Affiliations:** 1 Department of Surgery, Tohoku University Graduate School of Medicine, 1-1 Seiryou-machi, Aoba-ku, Sendai, Miyagi 980-8574 Japan; 2 Department of Lower Gastrointestinal Surgery, Kitasato University School of Medicine, 1-15-1 Kitasato, Minami-ku, Sagamihara, Kanagawa 252-0374 Japan; 3 Division of Cardiovascular Surgery, Tohoku University Graduate School of Medicine, 1-1 Seiryou-machi, Aoba-ku, Sendai, Miyagi 980-8574 Japan

**Keywords:** Left ventricular assist device, Colon cancer, Laparoscopic surgery

## Abstract

**Background:**

Left ventricular assist devices (LVADs) currently play an important role in the treatment of patients with end-stage heart failure who require a bridge to heart transplantation or destination therapy. With the development and improvement of the LVADs, the morbidity and mortality rates are declining and life expectancies increasing, and the number of patients with LVADs requiring non-cardiac surgery is likely to increase. We present the case of a patient with implantable LVAD who underwent laparoscopic right hemicolectomy for ascending colon cancer.

**Case description:**

The patient was a 66-year-old man who underwent LVAD implantation as a BTT 3 years prior. He suffered from severe anemia at follow-up, and a colonoscopy revealed ascending colon cancer. The LVAD pump was implanted in the epigastrium. The long C-shaped subfascial driveline tunnel was made, and driveline exit site was located on the left lateral abdominal wall. We assessed the positional relationship between the tumor and the driveline using X-ray and three-dimensional computed tomography (3D CT) images. 3D CT image allowed us to easily identify the location of the driveline, and we determined to perform laparoscopic right hemicolectomy. The port sites were decided upon carefully to avoid the driveline injury, and the driveline was marked on the skin before surgery. There were no adhesions in the abdominal cavity, and both the LVAD and the driveline were observable. The trocars were in nearly the same positions as in a standard laparoscopic right hemicolectomy. During the operation, the LVAD and the driveline did not interfere with the trocars. We successfully completed a standard laparoscopic right hemicolectomy despite hemorrhagic tendency. The patient was discharged without any bleeding complications during the postoperative course.

**Conclusion:**

Laparoscopic surgery is feasible and safe for patients with LVADs with intensive preoperative simulation and perioperative prevention of infection.

## Background

Left ventricular assist devices (LVADs) currently play an important role in the treatment of patients with end-stage heart failure who require a bridge to heart transplantation (BTT) or destination therapy (DT). LVADs are widely used to improve survival and quality of life in patients with severe heart failure. Because the number of patients with LVADs is increasing, the average wait time for heart transplantation is growing longer [1]. In addition, older patients with LVADs are increasing in number as the number of indications for DT expands [2]. Elderly patients with LVADs have the risk of malignant tumor development. Therefore, the number of patients with LVADs requiring surgery for abdominal cancer is also likely to increase.

The LVAD pump was implanted in the epigastrium. In some cases, a driveline that passed the subcutaneous tunnel was made, and penetrated the skin on the left lateral abdomen. Surgeons must be careful not to injure them during abdominal surgery. Here, we report a successful case of laparoscopic right hemicolectomy for a patient with LVAD.

## Case description

The patient was a 66-year-old man who underwent LVAD (HeartMate II, Abbott, Abbott Park, IL) implantation for ischemic cardiomyopathy as BTT 3 years prior. A blood test during a routine follow-up revealed severe anemia with hemoglobin level of 6.1 mg/dl. A colonoscopy revealed advanced ascending colon cancer. We diagnosed the cause of severe anemia in this patient as bleeding from the ascending colon cancer. Further investigation revealed no other causes of hemorrhage and no distant metastases. After discussions with cardiovascular surgeons and gastroenterologists, we concluded that the patient needed colectomy to treat his severe anemia. We planned colectomy for the ascending colon cancer with cardiovascular surgeons and anesthesiologists.

**Fig. 1:**
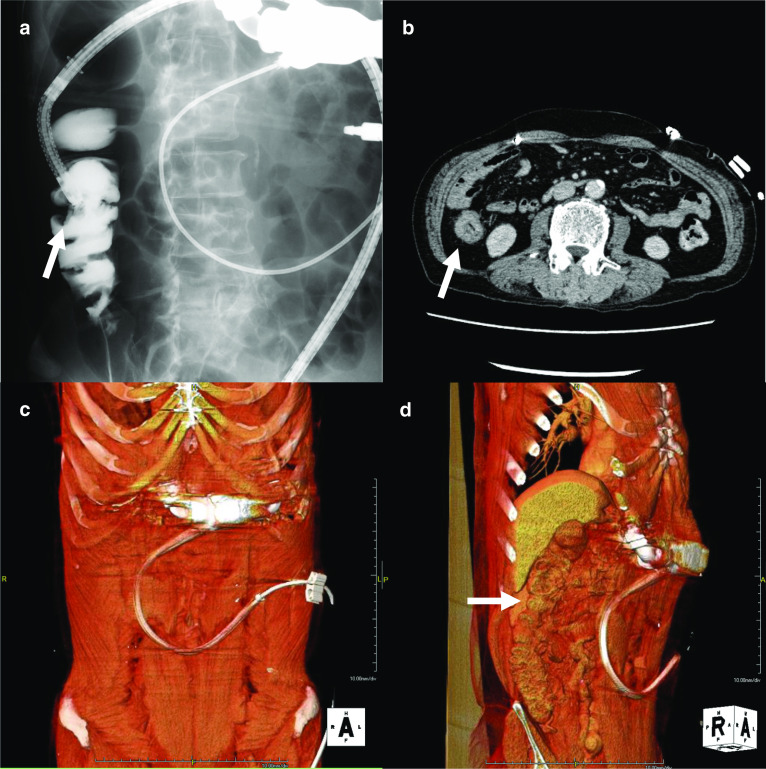
The positional relationship between the tumor and the driveline by X-ray (**a**) and CT images (**b**). The tumor (arrow) is away from driveline. **c**, **d** The positional relationship between the tumor (arrow) and the driveline by 3D CT images

The LVAD pump was implanted in the epigastrium. The long C-shaped subfascial driveline tunnel was made, and driveline exit site was located on the left lateral abdominal wall. In the case of laparotomy, driveline repositioning surgery was considered if necessary. The positional relationship between the tumor and the driveline was evaluated using X-ray and three-dimensional computed tomography (3D CT) images, and we decided that we place the trocars in nearly the same positions as in a standard laparoscopic right hemicolectomy (Fig. 1). Furthermore, in case the need to convert to open surgery arose, given the presence of a driveline across the lower abdomen, we also simulated driveline repositioning to the right side of the abdomen. Because the patient received anti-coagulation therapy with aspirin and warfarin, these drugs were interrupted at one week prior to surgery, and that with intravenous heparin infusion was started to maintain an activated partial thromboplastin time of 50–70 s.

**Fig. 2:**
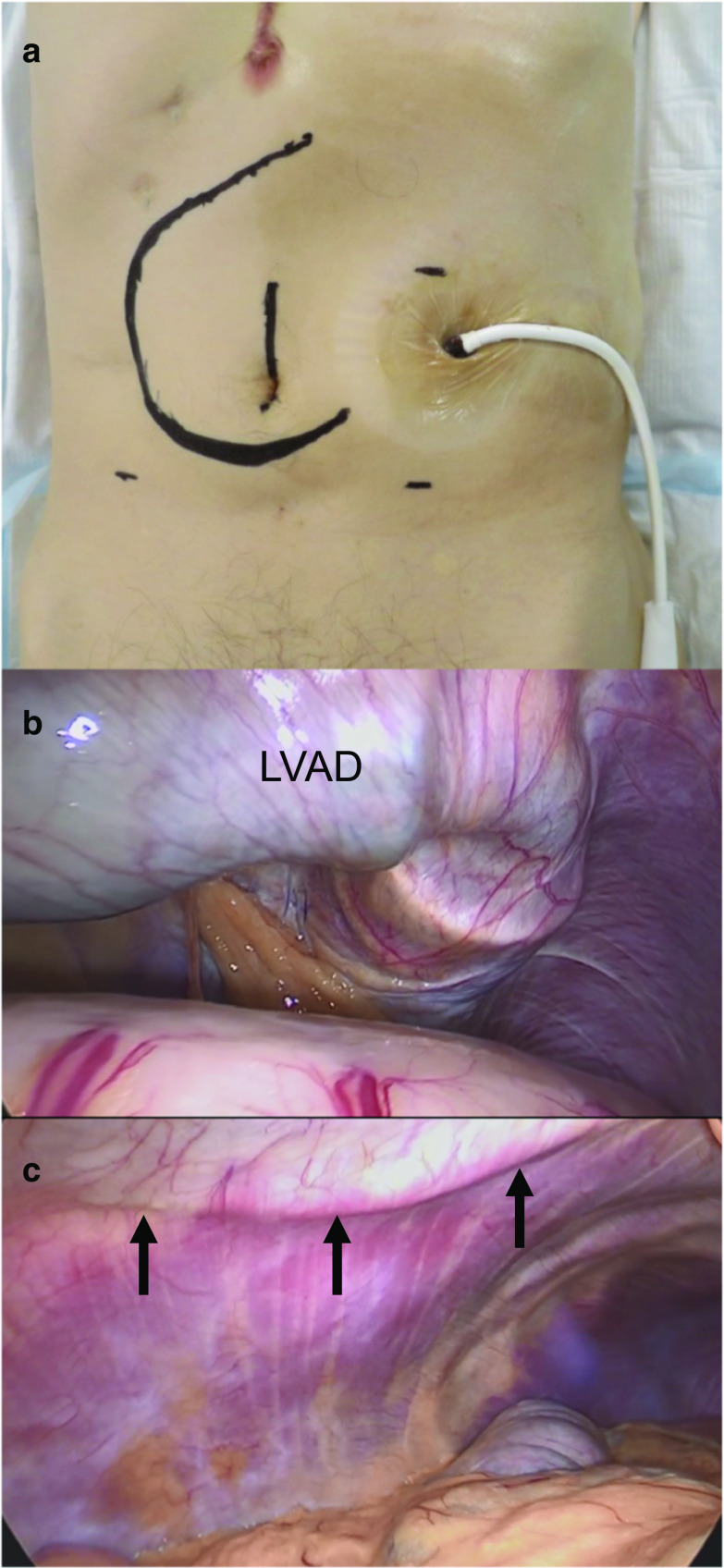
Preoperative marking (**a**) and laparoscopic view of the LVAD and the driveline (**b**, **c**). **a** The marking shows the driveline, the trocar positions and the small incision. **b** No adhesions were observed in the abdominal cavity even underneath the LVAD pump. **c** The driveline (black arrow) was relatively close to the midline and away from the trocar insertion positions of the right abdomen

**Fig. 3:**
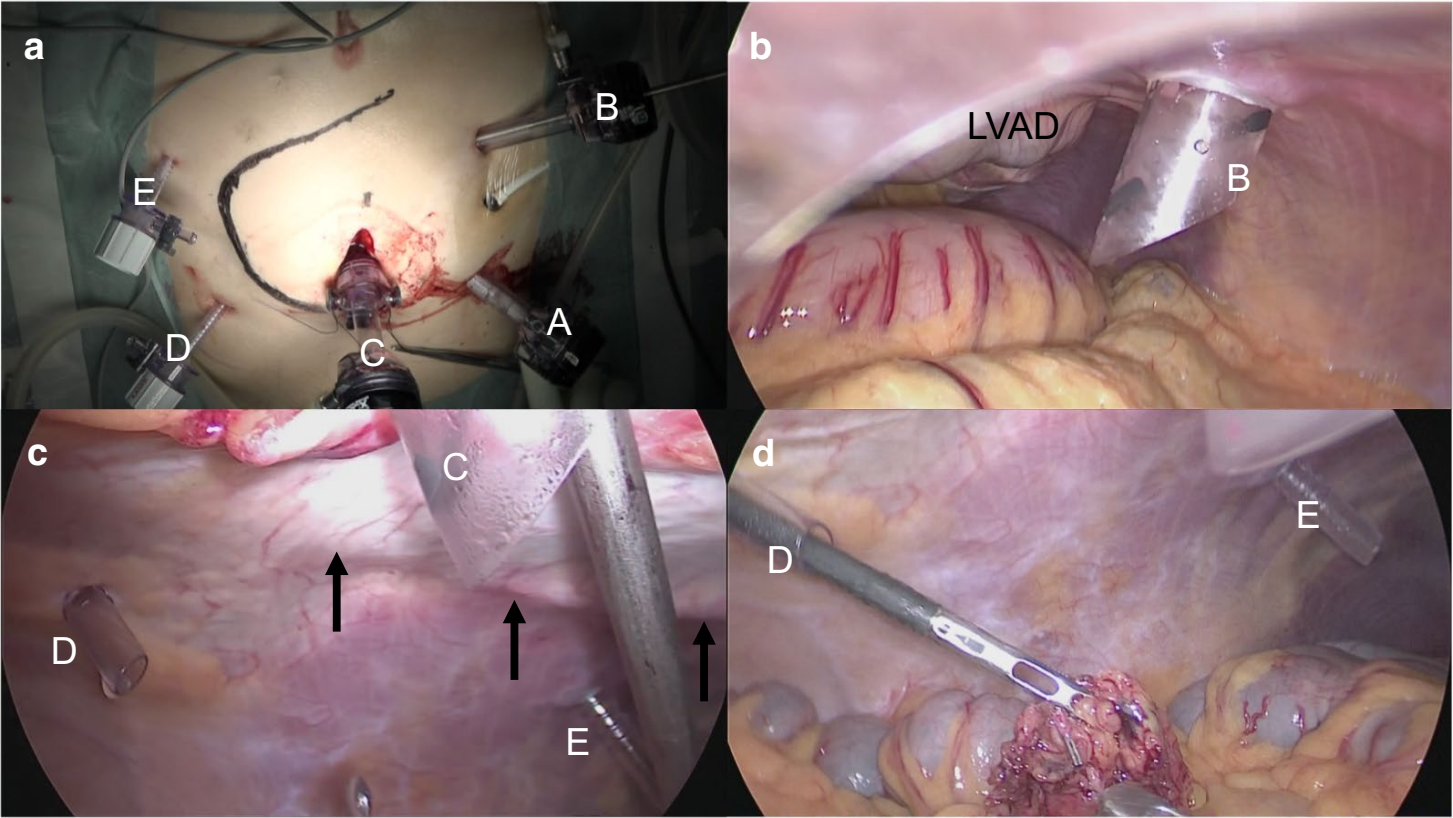
The trocar insertion sites from the body surface (**a**) and the abdominal cavity (**b**, **c**, **d**). **A** 12-mm port for the telescope; **B** 12-mm port for the surgeon’s right-hand device; **C** 12-mm blunt port for the surgeon’s left-hand device; **D**, **E** 5-mm ports for the assistant’s devices. All ports were inserted away from the driveline (black arrow) and the LVAD

The location of driveline was marked on the skin by a cardiovascular surgeon prior to surgery, and the port sites were decided upon carefully to avoid injury to the driveline due to trocar insertion (Fig. 2a). The first trocar was inserted using Hasson open technique via the umbilicus. Abdominal insufflation was initiated slowly and carefully because it has been reported pneumoperitoneum alters LVAD flow [3]. A pneumoperitoneum was done at a pressure of 10 mmHg, and a sufficient operative field was obtained without altering the hemodynamics. Upon observing the inside of the abdominal cavity, we found the LVAD and the driveline were observable without severe adhesions (Fig. 2b, c). The trocars were carefully inserted checking both the driveline observed from the abdominal cavity and the mark of the driveline on the abdominal wall. Two 12-mm trocars were inserted into the left abdomen, and two 5-mm trocars were inserted into the right abdomen, respectively (Fig. 3). The position of each trocar was nearly the same as the site marked before surgery and as a standard laparoscopic right hemicolectomy. The intraoperative position of the patient was basically in the reverse Trendelenburg position and temporally in the Trendelenburg position. As the reverse Trendelenburg position reduces venous return and the Trendelenburg position increases venous return, it was vital that changing of the operative position was performed slowly. Consequently, such actions led to minimal variation in the hemodynamics. During the operation, the LVAD and the driveline did not interfere with any trocars. Despite hemorrhagic tendency, we were able to perform laparoscopic right hemicolectomy uneventfully. The operating time was 204 min, and the amount of blood loss was 119 ml.

Intravenous heparin infusion was re-started 24 h after the operation was completed. Aspirin and warfarin were re-started orally on postoperative day (POD) 2. Because of high serum CRP level on POD3, the antibiotics were administered for 5 days until the patient’s serum CRP level was nearly normalized. A superficial incisional surgical site infection (SSI) was observed at the umbilical incision site on POD 5, but improved within a few days by washing alone. After oral warfarin management, he was discharged on POD 19 without any bleeding complications during the postoperative course. The histopathological examination showed that the tumor was classified as T3N2aM0 Stage IIIB (TNM classification, 8th ed.). We recommended adjuvant chemotherapy, but the patient refused due to social issues.

## Discussion

LVADs are implanted in patients with severe heart failure as BTT or DT. Because LVAD implantation improves survival and quality of life, DT accounts for approximately half of all LVAD implantation according to the Intermacs Database [2]. In addition, cases of long-term survival exceeding 5 years after LVAD implantation have been observed, and the number of cancer patients with LVADs is increasing [4]. Therefore, the number of patients with LVADs requiring surgery for abdominal cancer is likely to increase. Laparoscopic surgery in patients with LVADs has been reported in various types of abdominal surgery and more recently in bariatric surgery. However, reports of laparoscopic colectomy for LVAD patients with cancer are limited [3, 5–7]. It has also been recognized that, in patients with LVADs, the presence of the pump and the driveline in the abdominal wall necessitates the careful positioning of the trocars during laparoscopic surgery. Nevertheless, these reports do not show how the trocar positions were determined or how the pump and the driveline were inspected from the abdominal cavity.

In patients with certain types of LVAD, a pump is implanted in the epigastric region. A driveline is also implanted that is subcutaneously looped across the abdomen, as in the present case. In the case of laparotomy, the choice of an incision is technically difficult due to the pump and the driveline [8]. However, laparoscopic surgery is possible given that the trocar positions do not interfere with the pump and the driveline. In addition, a small incision is often made in the umbilicus and it is less likely to interfere with the pump and the driveline. Therefore, laparoscopic surgery is probably more useful than open surgery in the patients with LVAD. In laparoscopic right colectomy, as in the present case, the operator's ports are often on the left or lower abdomen and less likely to face interference from the pump or the driveline. On the other side, in laparoscopic left colectomy, the port sites may be limited by the driveline because the operator’s ports are often on the right abdomen. Therefore, careful preoperative simulation is considered necessary in laparoscopic left colectomy.

In the present case, we assessed the location of the driveline using 3D CT imaging prior to surgery. This imaging method was useful because it allowed us to easily identify the location of the driveline. There are two important points about the positional relationship between the driveline and trocars. First, interference between the driveline and the operator’s port may affect the surgical operation. Recently, laparoscopic surgery in patients with LVADs has been reported in various abdominal surgeries, especially in bariatric surgery. These reports discuss little about trocar positions and operative manipulation as, unlike cancer surgery, such operations require minimal vessel ligations or mobilization of the gastrointestinal tracts. However, Ishida et al. reported that changing the trocar positions from its usual positions may make surgical manipulation difficult [9]. Consequently, preoperative simulation should be performed sufficiently in upper abdominal surgeries with vessel ligations or mobilization of the gastrointestinal tracts. In the present case, performing a preoperative 3D CT image indicated the closeness of the left and right upper abdominal trocars to the driveline. We assessed that the left upper abdominal operator’s port could be inserted in our usual position. In fact, the trocar could be inserted in the same position as usual and did not interfere with the operation. However, if there is a need to alter the trocar positions, then such a change should be determined by preoperative simulating the surgical manipulation. Second, incisions near the pump or the driveline should be avoided because injury of these components can be fatal. The risk of infection of these components is related to how close they are to the incision [10]. Previous reports do not mention the distances between the trocar insertion sites and the driveline [3, 5–7]. By using 3D CT imaging to measure these distances, it is possible to avoid the trocar insertion sites close to the driveline. Laparoscopic surgery may be converted to open surgery in cases of bleeding or severe adhesions. In conversion cases from laparoscopic to open surgery, the driveline may need to be repositioned. Therefore, simulation for conversion to laparotomy should be performed before surgery. The 3D CT image, which makes it easy to identify the location of the driveline is useful for simulating conversion to laparotomy. In the present case, we simulated repositioning of the driveline to the right side of the abdomen due to the presence of the driveline across the lower abdomen. In cases where the driveline does not cross the lower abdomen, repositioning of the driveline would not be necessary.

Prevention of infection or bleeding is important in patients with LVADs. With regard to infectious complications, surgeons need to pay attention to SSI, because, if infection occurs, early intervention is necessary to avoid infection of the pump or the driveline. Laparoscopic surgery may reduce the risk of infection in comparison to open surgery and may therefore be useful for LVAD patients [11]. In the present case, superficial SSI was observed at the umbilicus incision. Fortunately, superficial SSI was observed at the umbilicus incision, yet did not spread to the site of the driveline or pump pocket due to early drainage and washing. With regard to bleeding complications, it is known that LVADs induce acquired von Willebrand syndrome [12, 13]. Therefore, surgeons need to be aware that the patients with LVADs tend to bleed even after anti-coagulation therapy is stopped.

## Conclusions

In conclusion, laparoscopic surgery for LVAD patients with cancer is feasible and safe with intensive preoperative simulation and prevention of infection. However, the therapeutic challenges of cancer remain. In the future, it is expected that the number of patients with advanced cancer will also increase. Therefore, it is necessary to establish therapeutic strategies for patients with advanced cancer who are fitted with an LVAD, including chemotherapy, and screening methods for the early detection of cancer.

## Data Availability

Not applicable.

## Abbreviations

LVAD: Left ventricular assist device
BTT: Bridge to heart transplantation
DT: Destination therapy
3D CT: Three-dimensional computed tomography
POD: Postoperative day
SSI: Surgical site infection
